# The Safety and Efficacy of Tofacitinib in 24 Cases of Pediatric Rheumatic Diseases: Single Centre Experience

**DOI:** 10.3389/fped.2022.820586

**Published:** 2022-02-08

**Authors:** Mikhail M. Kostik, Rinat K. Raupov, Evgeny N. Suspitsin, Eugenia A. Isupova, Ekaterina V. Gaidar, Tatyana V. Gabrusskaya, Maria A. Kaneva, Ludmila S. Snegireva, Tatyana S. Likhacheva, Rimma S. Miulkidzhan, Artem V. Kosmin, Anastasia V. Tumakova, Vera V. Masalova, Margarita F. Dubko, Olga V. Kalashnikova, Ivona Aksentijevich, Vyacheslav G. Chasnyk

**Affiliations:** ^1^Hospital Pediatry, Saint-Petersburg State Pediatric Medical University, Saint-Petersburg, Russia; ^2^Saint Petersburg State Health Care Establishment the City Hospital No 40 of the Resort District, Saint-Petersburg, Russia; ^3^Laboratory of the Molecular Oncology, N.N. Petrov's Institute of Oncology, Saint-Petersburg, Russia; ^4^Inflammatory Disease Section, National Human Genome Research Institute, Bethesda, MD, United States

**Keywords:** juvenile idiopathic arthritis, juvenile dermatomyositis, tofacitinib, JAK-inhibitors, interferonopathy, interferon type-I, alopecia

## Abstract

JAK-inhibitors are small molecules blocking the JAK-STAT pathway that have proven effective in the treatment of different immune-mediated diseases in adults and juvenile idiopathic arthritis (JIA).

**Aim of Study:**

To evaluate the safety and efficacy of tofacitinib in children with different rheumatic diseases.

**Material and Methods:**

We extracted information from 24 children with the following diagnosis: JIA (*n* = 15), undifferentiated systemic autoinflammatory diseases (SAIDs) (*n* = 7), and juvenile dermatomyositis (JDM) (*n* = 2) who have been treated with tofacitinib for a period of longer than 6 months. The treatment outcomes were classified according to the opinion of the attending physicians as having a complete response (CR), i.e., the absence of disease activity, or a partial response (PR)—a significant improvement of symptoms and disease activity, or no response (NR)—no changes in disease activity.

**Results:**

CR was achieved in 10/24 patients; 7/15 among JIA patients, 1/2 among JDM patients, 4/7 among SAID patients, and PR in 5/15 of JIA, 1/2 of JDM, and 3/7 of SAID patients. Three non-responders with JIA discontinued tofacitinib. Corticosteroids were successfully tapered off in 11/14 patients and discontinued in 2/14 patients. Four patients had side effects not requiring treatment discontinuation: liver enzyme elevation (*n* = 2), hypercholesterolemia (*n* = 1), lymphadenitis (*n* = 1).

**Conclusion:**

JAK-inhibitors are effective new therapies for the treatment of multiple immune-mediated diseases. Our experience has shown the best results in patients with JIA and JIA-associated alopecia, and type I interferonopathies. More data from randomized controlled clinical trials are needed to use JAK-inhibitors safely in pediatric rheumatic diseases.

## Introduction

A JAK-STAT signaling pathway is involved in the regulation of cell proliferation and differentiation, apoptosis, and immune signaling (1). Dysregulation in the JAK-STAT pathway has been implicated in the pathogenesis of a number of diseases: infections and sepsis, chronic arthritis, inflammatory bowel disease, multiple sclerosis, tumors, and others (2, 3). Therapies targeting the JAK-STAT pathway have been promising in the treatment of various immunological and inflammatory conditions (3). The success of the use of kinases-blockers in oncology (4) has paved the way for the use of JAK-inhibitors in rheumatic diseases, especially in rheumatoid arthritis: ruxolitinib (5), tofacitinib (6) and baricitinib (7). Food and Drug Administration (FDA) has approved tofacitinib for the treatment of rheumatoid arthritis (8), psoriatic arthritis, ulcerative colitis, and since 2020 for polyarticular juvenile idiopathic arthritis^1^ There are data from RCT in JIA available (9). Clinical trials of JAK-inhibitors for systemic lupus erythematosus, dermatomyositis, alopecia areata, atopic dermatitis, Crohn's disease, and uveitis are still ongoing (10). The indications of JAK-inhibitors for pediatric rheumatic diseases other than juvenile idiopathic arthritis, the optimal dosage, and safety have not been fully evaluated and recommendations are still pending.

## Patients and Methods

### Ethics

The Ethic Committee of Saint-Petersburg Sate Pediatric Medical University approved the study (protocol # 1/3 or 11.01.2021). Written consent of legal representatives for inclusion of the data and using of the pictures was obtained.

Attending physicians who participated in patients' treatment and evaluations are all pediatric rheumatologists with over 10 years of experience in this field.

### Patient Recruitment

The clinical and laboratory data from 24 children, who had been treated with tofacitinib for longer than 6 months were included in this retrospective case series study. Patients were divided into three groups: juvenile idiopathic arthritis (JIA), juvenile dermatomyositis (JDM), and undifferentiated systemic autoinflammatory diseases (SAIDs). Indications for tofacitinib were persistently high disease activity despite the treatment with corticosteroids and/or biologics treatment in most cases. In JIA patients the disease activity was assessed with calculation of JADAS-71 (11). Whole exome sequence (WES) and an interferon signature score were performed in 14/24 children (all children with systemic autoinflammatory disease (*n* = 7), children with JDM (*n* = 2) and sJIA (*n* = 4), and one with polyarticular JIA (multiple autoimmune features). An IFN-signature was measured by real-time PCR quantitation of five IFN I-regulated transcripts (*IFI44, IFI44L, IFIT3, LY6E, MX1*); median expression of ≥ 2 units was considered as a threshold. The treatment outcome was classified according to the opinion of the attending physicians as having a complete response (CR) i.e., the absence of disease activity (for JIA as defined by Wallace criteria) (12), or a partial response (PR)—a significant improvement of symptoms and disease activity (at least 50% improvement in active joints, ESR (erythrocyte sedimentation rate), CRP (C-reactive protein), VAS (visual analog scale) for JIA; improvement in muscle strength, skin rashes, and normalization of the muscle enzymes, ESR, CRP for JDM; a decrease in the number, duration and intensity of fever and rash episodes, improvement of CRP, ESR at least on 50%), or no response (NR)—no changes in disease activity.

### Statistics

Statistical analysis was performed with the software STATISTICA, version 10.0 (StatSoft Inc., USA). All continuous variables were checked by the Kolmogorov-Smirnov test, with no normal distribution identified. Continuous variables are presented as median and interquartile ranges (IQRs). Categorical variables are presented as proportions. Missing data were not imputed or included in the analyses. A comparison of two dependent quantitative variables was carried out using the Wilcoxon test. *P* < 0.05 was considered statistically significant.

## Results

The safety and efficacy of treatment with tofacitinib were analyzed in three separate groups: JIA, JDM and SAIDs.

### Juvenile Idiopathic Arthritis

The main indications for tofacitinib were inefficacy of previous biologic treatment (etanercept, adalimumab, abatacept, tocilizumab, canakinumab), presence of alopecia, and a strong desire of parents and patients to treat alopecia due to psychosocial distress. The data about baseline characteristics on 15 patients with JIA and changes in their disease activity during tofacitinib treatment are shown in Tables 1, 2.

**Table 1:** JIA patients: baseline characteristics and clinical course.

**JIA baseline characteristics**	**Results**		
Sex: girls/boys, n (%)	12 (80) / 3 (20)		
JIA onset age, years, median (IQR)	4.0 (2.9; 7.1).		
Uveitis, n (%)	2 (13.3)		
Rheumatoid factor positivity, n (%)	2 (13.3)		
Disease duration, years, median (IQR)	6.8 (3.7; 11.8)		
Time to tofacitinib, years, median (IQR)	6.0 (1.9; 10.2)		
Age of tofacitinib initiation, years, median (IQR)	12.0 (8.5; 14.5)		
Duration of tofacitinib treatment, years, median (IQR)	1.9 (0.8; 3.2)		
JIA characteristics	Before tofacitinib	Last visit	*p*
Active joints, median (IQR)	3.0 (1.0; 8.0)	0.0 (0.0; 4.0)	0.013
CRP, mg/l, median (IQR)	0.0 (0.0; 6.2)	0.0 (0.0; 0.0)	0.069
ESR, mm/h, median (IQR)	2.0 (0.0; 1.0)	1.0 (0.0; 0.0)	0.069
PGA-VAS, cm, median (IQR)	4.6 (4.1; 5.2)	1.3 (0.0; 3.9)	0.008
VAS physician, cm, median (IQR)	3.1 (1.8; 3.2)	0.5 (0.0; 2.1)	0.003
JADAS-71	15.0 (8.2; 17.2)	1.3 (0.0; 8.3)	0.003
Corticosteroid treatment, n (%)	5 (33)	4 (26.7)	0.109
Corticosteroids, mg/kg, median (IQR)	0.25 (0.11; 0.5)	0.05 (0.00; 0.35)	0.003

*JIA, juvenile idiopathic arthritis; cm, centimeters, CRP, C-reactive protein; ESR, erythrocyte sedimentation rate; IQR, interquartile range; Me, median, PGA-VAS, patient global assessment visual analog scale; JADAS-71, juvenile arthritis disease activity score*.

**Table 2:** Treatment modalities in patients with juvenile idiopathic arthritis.

**#**	**Sex/age of TOF initiation (years)**	**JIA subtype**	**Previous treatment**	**Current treatment**	**TOF dosage, mg/kg**	**TOF duration, mo**	**Efficacy**
1	F/14	Poly	MTX, ETA, TCZ, ADA	TOF, TCZ	0.27	22	PR
2	F/9	Poly (RF+) alopecia	CS, ETA, ABC, TCZ, ADA	lCS, TOF	0.3	37	CR
3	F/14	Poly	MTX, ABC, ETA	TOF	0.25	38	CR
4	F/16	Poly alopecia	MTX, ETA	TOF	0.15	31	CR
5	F/17	Poly	MTX, INX, ETA, ADA, TCZ	TOF	0.2	24	PR
6	F/14	ERA	MTX, ETA, ADA, TOF	SEC	0.25	21	PR
7	F/17	Poly	MTX, ADA, TCZ, ETA	TOF	0.15	39	CR
8	M/8	Poly	MTX, ADA, TCZ, TOF	GOL	0.25	6	NR
9	F/10	Poly (RF+)	MTX, CsA, TCZ, ETA, ABC	TOF	0,4	8	CR
10	F/11	Oligo alopecia	lCS, MTX	TOF	0.25	8	CR
11	F/8	Poly	ETA, TCZ, ADA	MTX, TOF	0.5	11	PR
12	F/15	Systemic	hCS, MTX, TCZ, CAN, ETA, TOF	lCS, RTX	0.5	38	NR
13	M/10	Systemic	hCS, TCZ, ABC, CAN, ETA	TOF, TCZ	0.4	23	PR
14	F/12	Systemic	hCS, MTX, CsA, ANA, TCZ, CAN	TOF, CAN	0.25	10	CR
15	M/4	Systemic	hCS, CAN, TOC	lCS, TOF, CAN	0.5	6	PR

*ABC, abatacept, ADA, adalimumab, ANA, anakinra, CR, complete response, CS, glucocorticosteroids (h-high dose, l-low dose), ERA, enthesitis-related arthritis, ETA, etanercept, GOL, golimumab, INX, infliximab, JIA, juvenile idiopathic arthritis, MTX, methotrexate, NR, no response, poly, polyarthritis, PR, partial response, RF, rheumatoid factor, RTX, rituximab, SEC, Secucinumab, TCZ, tocilizumab, TOF, tofacitinib*.

### Patient Characteristics

Patients had moderate-severe JIA; the JADAS-71 ranged from 4.5 to 27.7, most of the patients had JADAS-711 > 0 (63.6%). The number of preceding biologics were: four biologics in 3 patients (20%), three biologics in 8 (53.3%), two biologics in 1 (6.7%), one biologic in 2 (13.3%) Table 2. Two patients were receiving a combination of tofacitinib and methotrexate. Corticosteroid dose was successfully tapered. Four patients received a combined treatment of tofacitinib with biologics: two sJIA with canakinumab and two patients (one systemic and one polyarticular) with tocilizumab. In patient 1 and patient 13, the previous treatment with tocilizumab and tofacitinib alone was ineffective, and after adding tofacitinib to tocilizumab, patients had a better outcome compared to the previous treatment arms. After achieving the remission in patient 13, the interval between tocilizumab was extended to 6 weeks, and tofacitinib dose decreased from 10 mg two times a day (BID) to 5 mg BID. In two sJIA patients (patient 14 and patient 15) tofacitinib was added to canakinumab due to the relapse of macrophage activation syndrome and the inability to taper corticosteroids. In all sJIA patients, clinical whole exome sequencing (WES) did not revealed known gene variants associated with auto-inflammatory conditions.

### Treatment Outcomes

Seven patients (46.7%) achieved complete response (remission according to Wallace criteria), 5 patients (33.3%) had improvement (PR), and 3 (20%) patients were non-responders (NR), and tofacitinib was subsequently discontinued. Twelve patients have continued on tofacitinib. Patient 7 had a partial response, and tofacitinib was switched for an IL-17 inhibitor secukinumab.

### Juvenile Idiopathic Arthritis With Total Alopecia

We identified three JIA patients with total alopecia. All patients had autoimmune thyroiditis; however, thyroxine replacement therapy did not influence alopecia, neither did the long-term use of topical corticosteroids.

**Figure 1:**
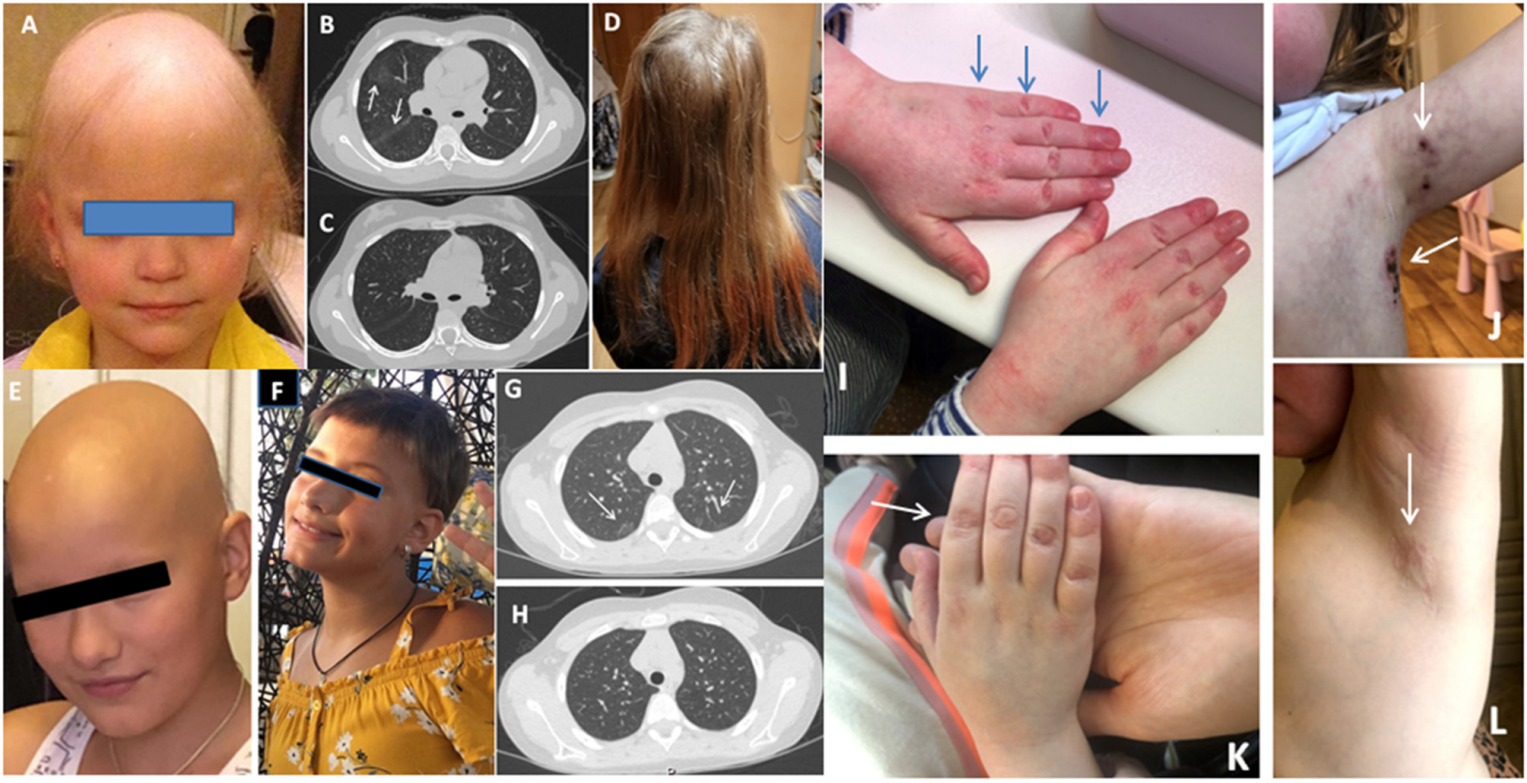
**(A)** Alopecia in JIA2 patient (before tofacitinib); **(B)** Chest CT from patient JIA2 (interstitial thickness and ground-glass opacities - arrows); **(C)** Chest CT from patient JIA2 following 3 years of tofacitinib therapy (resolution of interstitial thickness and ground glass opacities); **(D)** Hair growth in JIA2 patient (3years on tofacitinib); **(E)** Alopecia in patient JIA10 before tofacitinib treatment and after 6 months of treatment **(F)**; **(G)** Ground-glass opacities in patient JDM1 before tofacitinib (arrows) and 2 years on tofacitinib **(H)**; **(I)** The hands with Gottron papules before tofacitinib treatment in JDM2 patient and the left axillary area (arrows) with necrotizing changes **(J)**; **(K)** the right hand 16 weeks after tofacitinib treatment and the left axillary area **(L)** after 6 months of tofacitinib treatment of the same patient.

Patient JIA2 has had severe RF (+) polyarthritis, alopecia subtotalis (Figure 1A), a single uveitis episode, autoimmune thyroiditis, and interstitial lung disease (Figure 1B). Her RF was more than 7,700 IU/ml (n.v. <20 IU/ml). She did not achieve remission on etanercept, adalimumab, abatacept, tocilizumab and required treatment with corticosteroids. Her alopecia was steroid-dependent. After the tofacitinib treatment with 1.25 mg of prednisolone every other day her arthritis has been under control. She has had a complete resolution of lung disease (Figure 1C) on a chest CT scan, and she has had remarkable hair growth (Figure 1D). Clinical exome sequencing detected one rare variant of unknown clinical significance (VUS) *IL1RN:* NM_173841: c.10G>C; p.A4P and a likely benign variant in the *MEFV* gene*: NM_000243.3:* c.1105C>T; p.P369S.

Patient JIA4 has had RF-negative polyarthritis since the age of 4 years and developed alopecia areata at the age of 13 following 5 years of treatment with etanercept and methotrexate. She did not respond to scalp steroid injection, she had persistent arthritis, and tofacitinib was initiated after excluding lupus-like syndrome. She required an increased dose of tofacitinib from 5 mg two times every day (BID) to 5 mg three times every day (TID).

Patient JIA10 had oligoarthritis and alopecia areata since the age of 4 years. After 4 years of remission and methotrexate discontinuation, she presented with arthritis flare, developed alopecia totalis, and autoimmune thyroiditis, and both conditions were steroid-dependent (Figure 1E). Treatment with tofacitinib 5 mg BID was started to limit her alopecia and arthritis with complete resolution of both conditions (Figure 1F).

### Juvenile Dermatomyositis

In this cohort of patients, we identified only two cases with juvenile dermatomyositis (JDM). Patient JDM1 is an 8-year-old girl who presented with severe muscle (CMAS=10) and skin involvement. She was treated with methylprednisolone pulse therapy, intravenous immunoglobulin, prednisone 2mg/kg, and methotrexate 15 mg/m^2^/week. Her muscle strength has completely recovered in 3 months, but her skin rash deteriorated on steroid tapering. Mycophenolic acid, followed by methotrexate and topical agents were used without efficacy. Interstitial lung disease was diagnosed on CT 2 years after the disease onset (Figure 1G). Tofacitinib was initiated 2 years after the disease onset. Her facial erythema improved however, Gottrone's papules did not disappear. Ground glass opacities disappeared (Figure 1H). Therapy with tofacitinib allowed for tapering prednisone to 0.1 mg/kg. Her IFN-scores before tofacitinib and one year after the treatment were 33.75 U and 10.25 U (nv <2 U), respectively. Whole exome sequence detected a non-sense variant in the Leukocyte Tyrosine Kinase (*LTK)* gene: NM_002344.6: c.1393_1394del; p.P465^*^ (rs780209477) inherited from her asymptomatic father, and VUS in the *IL21R* gene NM_181078.3: c.128A>C; p.H43P (rs757749249) inherited from her asymptomatic mother. Also, a rare VUS in *PTPN5:* NM_032781: c.1216G>A; p.E406K (rs139371305) was identified.

Patient JDM2 is a 6-year-old girl who has had JDM for 2 years presenting with muscle weakness, heliotrope rash, Gottron papules, livedo reticularis, and necrotizing skin changes above the knees, elbows, clavicula and in bilateral axillae (Figures 1I,J). Tofacitinib was initiated after one year from the disease onset with complete resolution of all symptoms (Figures 1K,L). Her IFN-scores before and 6 months after the treatment with tofacitinib were 10.8 U and 1.2 U (nv <2 U), respectively. Clinical exome sequence revealed a rare VUS in the *NLRP12* gene: NM_144687:c.154G>A; p.G52S (rs369053968). No variants in interferon-pathway related genes were identified. In both cases, tofacitinib allowed for tapering steroids and improved the disease's course.

### Systemic Autoinflammatory Diseases

Tofacitinib was prescribed to patients with autoinflammatory diseases in the following cases: i) patient has a genetically confirmed interferonopathy or disease likely mediated by STAT-pathway; ii) patient has clinical features of interferonopathy without genetic confirmation (early disease onset, recurrent fever, skin rash, lipoatrophy, neuropathy); iii) previous treatment with other biologics failed, and a literature search suggested the possibility of successful use of JAK-inhibitors. Patients' characteristics are shown in Table 3.

**Table 3:** The clinical characteristics and genetic findings in patients with SAIDs.

**#**	**Sex/age of TOF onset**	**Symptoms**	**Previous treatment**	**Current treatment**	**Duration**	**Efficacy**	**Rare DNA variants**
1	F/13	Severe inflammation, aortitis, colitis (Crohn's like), phlebitis	INX, TCZ, ADA	CS (0.1 mg/kg), TOF (0.5 mg/kg)	12	CR	*NOD2*;NM_022162: c.2578G>A; p.Ala860Thr; *NOD2*;NM_022162: c.2722G>C; p.Gly908Arg
2	F/7	Skin rash, recurrent inflammation, failure to thrive	CAN, TCZ	CS (0.1 mg/kg); TOF (0.5 mg/kg); CAN	38	PR (Episodes of fever)	None detected
3	F/15	CANDLE-like interferonopathy	ETA, RTX, CAN	CS (0.15 mg/kg); TOF (0.5 mg/kg)	43	CR	*PSMD5;* NM_005047.3: A splice-region variant
4	F/17	Systemic inflammation, nodular erythema, panniculitis, sialadenitis, hepatitis, headaches, growth failure. High IFN-score before tofacitinib initiation	AZA, MMF	CS (0.15 mg/kg); TOF (0.5 mg/kg)	10	PR; PR (Fever on prednisone tapering)	*IFIH1;*NM_022168: c.2035_20 36del; p.Leu679Ilefs*3 and c.1795delG; p.Val599FLeufs*10
5	F/12	Systemic inflammation, scleroderma/lupus like skin disease, pancytopenia, arthritis	TCZ	CS (0.1 mg/kg); TOF (0.5 mg/kg	21	PR	*STAT3;*NM_139276.2: c.1343A>C; p.Gln448Pro
6	F/9	Systemic inflammation, distal necrosis of finger, livedo, erythema, arthritis	CYC, ETA	CS (0.05 mg/kg); TOF (0.5 mg/kg)	19	CR	None detected
7	F/17	Systemic inflammation, panniculitis, polyneuropathy	hCS, CsA, ETA, IVIG	TCZ, TOF (0.3 mg/kg)	6	PR	*IFIHI;*NM_022168: c.1558A>G; p.Thr520Ala

Patient SAID1 is a 13-year-old girl from consanguineous marriage (second cousins). She initially presented with features suggestive of Crohn's disease and was successfully treated with corticosteroids, azathioprine, and mesalamine. After 1½ years, she suddenly developed fever, high inflammatory markers, and intensive lumbar pain. CT-angiography revealed severe aortitis, and she was diagnosed with Takayasu arteritis. Increased doses of corticosteroids and infliximab (10 mg/kg) improved her systemic inflammation, but after tapering of steroids to 1 mg/kg, the signs of disease re-appeared. Furthermore, the patient developed claudication and multiple phlebitis, she tested positive for the HLAB51 allele, and Bechet's disease was considered in differential diagnosis, although she did not have oral or genital ulcers. We started adalimumab in the induction regimen, but this treatment failed as well as therapy with tocilizumab followed by canakinumab. Whole-exome sequence revealed two rare variants in *NOD2*, one of which has been associated with susceptibility Crohn's disease (Table 3). The patient responded well only to an increased dose of tofacitinib (10 mg BID), while the initial dose5—mg BID was partially effective.

Patient SAID2 presented with clinical features (fever, extensive panniculitis, and severe systemic inflammation) suggesting an interferonopathy, at the age of 6 months. She presented at our clinic at the age 6 years with severe growth delay, failure to thrive, and signs of steroid toxicity (she received high doses of steroids throughout her life). Initial treatment was with canakinumab that was gradually increased from 4 mg/kg to 12 mg/kg (150 mg per injection every 4 weeks) with partial response; however, it allowed tapering methylprednisolone to 2–4 mg/day. Overall, this therapy did not suppress her disease activity. Initially, tofacitinib was not available but after 2 years and exacerbation of her symptoms on canakinumab, we added tofacitinib. The patient still had an active disease on 5 mg BID, while her symptoms were ameliorated on 5 mg TID without need to increase steroids. Her clinical WES did not reveal any pathogenic or likely pathogenic variants.

Patient SAID3 had a classical feature of interferonopathy, a CANDLE-like phenotype, with early disease onset, failure to thrive, recurrent fever, short stature, peripheral lipoatrophy, hepatomegaly, erythematosus, livedoid and nodular skin rash with ≪punched scars≫ formation, and necrotizing vasculitis. Before tofacitinib, she failed therapy with etanercept, rituximab, and canakinumab. She developed ischemic lesions with gangrene, severe Cushing's syndrome, dysmorphic features, panniculitis, and hepatosplenomegaly. On tofacitinib, she has had impressive clinical improvement, corticosteroids were tapered off to 0.15 mg/kg, and she additionally received IVIG for control of vasculitis. Her disease flared following COVID-19 infection. We increased the dose of tofacitinib from 5 mg BID to 10 mg BID with monthly IVIG. Her WES identified a novel putative splice site variant in the *PSMD5* gene that warrants further investigation. PSMD5 is a non-ATPase regulatory subunit that promotes 26S proteasome assembly (13).

Patient SAID4 had clinical features suggestive of interferonopathy: fever, inflammation, panniculitis, sialadenitis, nodular rash, hepatitis, migraine headaches, and growth failure. Her IFN-score was 11.25 U (nv <2 U) before tofacitinib initiation. Her previous treatment with corticosteroids and azathioprine or mycophenolate mofetil was ineffective, and she required high doses of corticosteroids. Treatment with tofacitinib for 3 months only partially controlled her disease (no fever flares, and rash) and allowed tapering corticosteroids to 0.15 mg/kg. Unfortunately, following tapering, her disease flared with highly increased CRP. Her IFN-score was still high at 6 months after the tofacitinib initiation (IFN-score 11.3). She was found to be a carrier for compound-heterozygous loss of function mutations in the *IFIH1* gene, encoding a cytoplasmic viral RNA receptor MDA5 that activates type I interferon signaling. The presence of both variants has been confirmed by Sanger sequencing. Pathogenic variants in this gene have been associated with Aicardi-Goutières syndrome-7 (AGS7) (14).

Patient SAID5 (female, 12 years) presented with scleroderma/lupus like disease (facial features, skin thickness), accompanied with fever, livedoid rash, impressive lymphadenopathy, pleuritis, pancytopenia, increased CRP>200 mg/l (nv <5). Initial treatment with steroids and tocilizumab was partially effective. Following a switch to tofacitinib, her disease was under control, and corticosteroids were given at a minimal dose, 0.1 mg/kg. Tofacitinib was used initially in the doses of 5 mg BID, but her dose was increased to 15 mg/day due to persistent moderate activity, which led to control of her disease activity. She was found to carry a novel VUS variant in *STAT3* NM_139276.2: c.1343A>C; p.Q448P. Heterozygous pathogenic variants in this gene have been associated with hyper IgE syndrome, however this patient did not present with immunodeficiency or eczema.

Patient SAID6 (female, 9 years) presented with features of polyarteritis nodosa (PAN): systemic inflammation, distal tip fingers and tongue necrosis, livedoid rash, erythema nodosum, and arthritis. She was treated with prolonged course of high dose steroids and cyclophosphamide at her local hospital. Deficiency of adenosine deaminase 2 (DADA2) was suspected and she was treated with etanercept for 6 months without efficacy. DADA2 was ruled out as her ADA2 enzymatic level was normal and clinical exome sequencing revealed no ADA2 pathogenic variants. She remained steroid-dependent and switching etanercept to tofacitinib led to complete control of her disease, and corticosteroids were discontinued.

Patient SAID7 is a 16-year-old-girl. She had fever, pancytopenia, petechial rash, hepatomegaly, edema of the lower extremities and systemic inflammation: CRP 201 mg/l, (n.v. <5), ferritin 300,000 ng/ml, (n.v. 13-150), ALT 80 U/l, (n.v. 55) and AST 324 U/l, (n.v. 34). Hematological malignances and autoimmune disorders were ruled out. She was initially treated with pulse methylprednisolone, cyclosporine A in her local hospital. She developed panniculitis nodules in the back, the abdomen and the lower extremities with muscle weakness. Pancytopenia, inflammation had recurred. Biopsy of nodules reveled adipocytes and xanthoma cells and infiltrating lymphocytes. The exudate of nodules contained neutrophils and was sterile. The further treatment with cyclosporine A and etanercept resolved fever and decreased the size and number panniculitis nodules, but cytopenia persisted. Her IFN-score was mildly elevated −5.5 U (nv <2). Cyclosporine A was stopped. Tofacitinib was started with panniculitis and rash resolving. Due to pancytopenia etanercept was switched for tocilizumab, and 6 months of combined treatment with tofacitinib and tocilizumab resolved all clinical and laboratory manifestations. A rare heterozygous variant in the *IFIH1* gene was identified NM_022168: c.1558A>G; p.T520A (rs145641024), however, current prediction algorithms disagree on the potential impact of this variant on the protein function, and thus this variant is defined as VUS.

### Overall Efficacy and Corticosteroid Tapering

Complete response was achieved in 10/24 (41.7%) patients; 5/12 (41.7%) among JIA patients, 1/2 (50%) among JDM patients, 4/7 (57.1%) among SAID patients. Partial response was achieved in 5/15 of JIA (33.3), 1/2 of JDM (50%) and 3/7 (42.9%) of SAID patients. There were three non-responders, both diagnosed with JIA.

During the observation period corticosteroids were successfully tapered in 11/14 (78.6%) patients and median dose of corticosteroids was reduced from 0.25 (0.2; 0.5) to 0.1 (0.05; 0.125) mg/kg (p=0.005) and discontinued in 2/14 (14.3%) patients, who received steroids before tofacitinib.

The median tofacitinib dose in JIA patients was 0.25 (0.2; 0.4) mg/kg, which was lower than in patients with primary interferonopathies (AID and JDM) −0.5 (0.5; 0.5) mg/kg (p=0.003).

### Safety

Four patients had side effects not requiring treatment discontinuation: liver enzymes elevation (*n* = 2), hypercholesterolemia (*n* = 1), lymphadenitis (*n* = 1). None has had severe infections, including no cases of new VZV infection were observed during the observation period.

## Discussion

JAK-inhibitors are new targeted small molecule based-therapies in pediatric rheumatology. They have shown efficacy in adult rheumatic conditions and are included in clinical guidelines of rheumatoid arthritis (15), psoriatic arthritis (16), ankylosing spondylitis (17). Currently, the experience of JAK-inhibitors in the pediatric population is scarce and limited to a single randomized placebo-controlled study in JIA and several studies in small groups of patients with immune-mediated diseases (18, 19). The largest randomized, double-blind, placebo-controlled withdrawal study of tofacitinib in JIA showed that patients with polyarticular course of JIA treated with tofacitinib have significantly lower rate of disease flare at 44 weeks compared to placebo-group (29% vs 52.9%, p=0.0031). JIA ACR30/50/70 response rates were higher in tofacitinib group (70.8% vs 47.1%, p=0.003; 66.7% vs 47.1%, p=0.017; 54.2% vs 37.1%, p=0.039) (20). In our group, 5/11 patients with non-systemic JIA had a complete response with 100% improvement in JADAS-71, while only 1 patient with ERA did not respond to tofacitinib and required a change in treatment. The remaining 5/11 patients had a partial response with a median improvement of JADAS-71 at 43.3%. Despite the ongoing disease activity, the treatment with tofacitinib was continued due to improved arthritis course and quality of life and lack of other options for disease control. JAK-inhibitors are recommended in patients with rheumatoid arthritis who don't respond to methotrexate (15, 21), but the place of JAK-inhibitors in the management of JIA has not been yet defined.

Adult-onset Still's disease (AOSD) and systemic JIA (sJIA) are two autoinflammatory diseases with similar clinical presentation and treatment strategies. Interleukin-1 and interleukin-6 cytokines contribute to disease pathogeneses, and anti-IL1 and anti-IL-6 biologics have comparable efficacy in the treatment of these patients (22). JAK-inhibitors block signal transduction downstream of many cytokine receptors, so theoretically they might be more efficacious to control sJIA than classical biologics, especially in the resistant cases (3). Seven of 14 patients (50%) with AOSD achieved complete remission in a Chinese single-center study, whereas partial response was noticed in 6 patients. Four patients discontinued treatment due to partial response or non-response and side effects (23). We have found in the literature only 2 cases of systemic JIA that were treated with JAK-inhibitors. The first is a 13-year-old girl with refractory steroid-dependent disease and six-month treatment with tofacitinib led to the resolution of systemic and articular symptoms and allowed for discontinuation of corticosteroid therapy (24). The second is a 6-year-old girl with fever, polyarthritis and rash who received ruxolitinib for 23 months. Previously, she did not respond to treatment with anakinra, tocilizumab, canakinumab, and infliximab. The efficacy was assessed as partial response, and corticosteroids were reduced from 3 mg/kg to 1 mg/kg on ruxolitinib treatment (25). In this report, we describe our experience with four patients with systemic JIA. Only in 1 case was this treatment ineffective, despite increasing doses up to 10 mg BID, neither this patient responded to previous treatments with multiple biologics. The remaining three patients were receiving combination treatments with biologics (2 patients – tocilizumab and 1 patient canakinumab) showed good clinical and laboratory control of the disease activity. Despite the possibility to block several cytokines, these two patients required combination treatment of tofacitinib with biologics.

The prevalence of alopecia in JIA is scarce. In the Italian cohort of 79 JIA patients one patient (1.3%) had alopecia (26), while in 3,510 patients with alopecia areata JIA was registered in 0.14% of cases (27). Several publications support the evidence of the efficacy of JAK-inhibitors in alopecia treatment. Ying-Xiu Dai et al., reported three cases of alopecia in children successfully treated with tofacitinib (28). In another study, more than 6 months of treatment with tofacitinib improved SALT (Severity of Alopecia Tool) in 9/11 of patients (29). Topical JAK-inhibitors might be another treatment option for alopecia areata. Half of six children with alopecia areata had hair overgrowth on topical JAK-inhibitors treatment (30). In our study, all three patients with JIA and alopecia had impressive hair growth and complete response to therapy regarding their arthritis.

The role of the IFN-I pathway has been reported in the pathogenesis of dermatomyositis. Overexpression of IFN-stimulated genes has been found in the muscle, skin and blood of patients with dermatomyositis. IFN-signature is considered as possible disease activity biomarker and flare predictor (31). Ding and colleges reported successful management of 24/25 children refractory JDM with JAK-inhibitors (18 patients were treated with ruxolitinib and seven patients with tofacitinib). Complete skin rash resolution was achieved in 67% cases, 28% of patients discontinued steroids, and 7/10 patients with decreased muscle strength had improved CMAS score (18). The prospective, open-label clinical trial of tofacitinib in 10 adults with refractory dermatomyositis demonstrated efficacy of JAK-inhibitors in all patients (32).

In our study, two patients with JDM had elevated IFN-score before tofacitinib. Patient JDM1 had a partial skin response without normalization of her IFN-score. Patient JDM2 had complete muscle and skin response with normalization of IFN-score level. The role of IFN-score and JAK-inhibitors in the management of JDM has not been defined, and further investigations are required.

Type I interferonopathy comprises autoinflammatory syndromes characterized by activation of type I interferon signaling. To date, there are more than 20 monogenic types of interferonopathies (33). Clinical classification criteria have been recently developed for interferonopathies, helping to select candidates for genetic testing (34). Baricitinib has been studied in patients with CANDLE (*n* = 10), SAVI (*n* = 4), and undifferentiated interferonopathy (*n* = 4). The remission was achieved in half the patients with CANDLE, but primary response measured by disease-specific daily score and corticosteroid response was reached in 12/18 and 10/14 of patients (19). The efficacy of baricitinib has been demonstrated in a case report of CANDLE syndrome (35) and several cases of SAVI, however to a lesser degree (36, 37). In case series including patients with Aicardi-Goutieres syndrome, which carry mutations in the *IFIH1* gene, the efficacy of JAK-inhibitors in ameliorating skin and systemic manifestations have been reported (38, 39).

The last review of P.G. Gomez-Arias and colleagues, including 24 patients with type I interferonopathies showed that JAK-inhibitors improved clinical symptoms and decreased the number of flares and inflammatory markers (40). Besides, an open-label study of baricitinib (LY3009104) in adult and pediatric Japanese participants with NNS/CANDLE, SAVI, and AGS is ongoing^2^.

Adverse events were observed in 153/225 (68%) children with JIA receiving tofacitinib, while serious adverse events were registered in seven patients. Discontinuations due to adverse events required 26 patients to discontinue this treatment in 26 (11.6%) (9).

Short half-life (3 hours) of JAK inhibitors compared to biologics with a longer half-life time, might be an additional benefit for children in preventing susceptibility to infections. In case of infection, the discontinuation of tofacitinib leads to rapid wash-out of medication from the blood (41). Other benefits for children who are destined for life-long therapies, include the lack of immunogenicity and oral administration (9, 42). We have to note about potential significant risks of using JAK inhibitors in combination with biologics and about the potential risks of using tofacitinib dose greater than 5mg two times a day.

## Conclusion

JAK-inhibitors are effective new therapies for the treatment of multiple immune-mediated diseases. Our experience has shown the best outcomes in patients with JIA and JIA-associated alopecia, and type I interferonopathies, compared to JDM, although we had observed only two patients diagnosed with JDM. More data from systematic real world studies are needed to assess the use of JAK-inhibitors safely in pediatric rheumatic diseases.

## Data Availability Statement

The original contributions presented in the study are included in the article/supplementary material, further inquiries can be directed to the corresponding author.

## Ethics Statement

The study involving human participants was reviewed and approved by the Ethic Committee of Saint-Petersburg State Pediatric Medical University (protocol # 1/3 from 11.01.2021). Written informed consent to participate in this study was provided by the participants' legal guardian/next of kin. Written informed consent was obtained from the minor(s)' legal guardian/next of kin for the publication of any potentially identifiable images or data included in this article.

## Author Contributions

MK, RR, ES, and IA designed the study and wrote the manuscript. MK, RR, ES, EI, EG, TG, MK, LS, TL, RM, AK, AT, VM, MD, OK, and VC collected and analyzed data. All authors contributed to manuscript revision, read, and approved the submitted version.

## Funding

The work was supported by Russian Science Foundation grant 20-45-01005.

## Conflict of Interest

The authors declare that the research was conducted in the absence of any commercial or financial relationships that could be construed as a potential conflict of interest.

## Publisher's Note

All claims expressed in this article are solely those of the authors and do not necessarily represent those of their affiliated organizations, or those of the publisher, the editors and the reviewers. Any product that may be evaluated in this article, or claim that may be made by its manufacturer, is not guaranteed or endorsed by the publisher.

## Footnotes

^1^ https://www.accessdata.fda.gov/drugsatfda_docs/label/2020/203214s026lbl.pdf

^2^ https://clinicaltrials.gov/ct2/show/NCT04517253

## References

[1] Xin P Xu X Deng C Liu S Wang Y Zhou X. The role of JAK/STAT signaling pathway and its inhibitors in diseases. Int Immunopharmacol. (2020) 80:106210. 10.1016/j.intimp.2020.106210 31972425

[2] O'Shea JJ Schwartz DM Villarino AV Gadina M McInnes IB Laurence A. The JAK-STAT pathway: impact on human disease and therapeutic intervention. Annu Rev Med. (2015) 66:311–28. 10.1146/annurev-med-051113-024537 25587654 PMC5634336

[3] Schwartz DM Kanno Y Villarino A Ward M Gadina M O'Shea JJ. JAK inhibition as a therapeutic strategy for immune and inflammatory diseases. Nat Rev Drug Discov. (2017) 16:843–62. 10.1038/nrd.2017.201 29104284

[4] Druker BJ Talpaz M Resta DJ Peng B Buchdunger E Ford JM et al. Efficacy and safety of a specific inhibitor of the BCR-ABL tyrosine kinase in chronic myeloid leukemia. N Engl J Med. (2001) 344:1031–7. 10.1056/NEJM200104053441401 11287972

[5] Verstovsek S Mesa RA Gotlib J Levy RS Gupta V DiPersio JF et al. A double-blind, placebo-controlled trial of ruxolitinib for myelofibrosis. N Engl J Med. (2012) 366:799–807. 10.1056/NEJMoa1110557 22375971 PMC4822164

[6] Fleischmann R Kremer J Cush J Schulze-Koops H Connell CA Bradley JD et al. Placebo-controlled trial of tofacitinib monotherapy in rheumatoid arthritis. N Engl J Med. (2012) 367:495–507. 10.1056/NEJMoa1109071 22873530

[7] Keystone EC Taylor PC Drescher E Schlichting DE Beattie SD Berclaz PY et al. Safety and efficacy of baricitinib at 24 weeks in patients with rheumatoid arthritis who have had an inadequate response to methotrexate. Ann Rheum Dis. (2015) 74:333–40. 10.1136/annrheumdis-2014-206478 25431052 PMC4316868

[8] Jamilloux Y El Jammal T Vuitton L Gerfaud-Valentin M Kerever S Sève P. inhibitors for the treatment of autoimmune and inflammatory diseases. Autoimmun Rev. (2019) 18:102390. 10.1016/j.autrev.2019.102390 31520803

[9] Ruperto N Brunner HI Synoverska O Ting TV Mendoza CA Spindler A et al. Tofacitinib in juvenile idiopathic arthritis: a double-blind, placebo-controlled, withdrawal phase 3 randomised trial. Lancet. (2021) 398:1984–96. 34767764 10.1016/S0140-6736(21)01255-1

[10] Fragoulis GE McInnes IB Siebert S. JAK-inhibitors. New players in the field of immune-mediated diseases, beyond rheumatoid arthritis. Rheumatology (Oxford). (2019) 58:i43–i54. 10.1093/rheumatology/key276 30806709 PMC6390879

[11] Consolaro A Ruperto N Bazso A Pistorio A Magni-Manzoni S Filocamo G et al. Development and validation of a composite disease activity score for juvenile idiopathic arthritis. Arthritis Rheum. (2009) 61:658–66. 10.1002/art.24516 19405003

[12] Wallace CA Giannini EH Huang B Itert L Ruperto N Childhood Arthritis Rheumatology Research Alliance. American College of Rheumatology provisional criteria for defining clinical inactive disease in select categories of juvenile idiopathic arthritis. Arthritis Care Res (Hoboken). (2011) 63:929–36. 10.1002/acr.20497 21717596

[13] Levin A Minis A Lalazar G Rodriguez J Steller H. PSMD5 inactivation promotes 26S proteasome assembly during colorectal tumor progression. Cancer Res. (2018). 78:3458–68. 10.1158/0008-5472.CAN-17-2296 29716915 PMC6030489

[14] Rice GI Del Toro Duany Y Jenkinson EM Forte GM Anderson BH Ariaudo G et al. Gain-of-function mutations in IFIH1 cause a spectrum of human disease phenotypes associated with upregulated type I interferon signaling. Nat Genet. (2014) 46:503–9. 10.1038/ng.2933 24686847 PMC4004585

[15] Smolen JS Landewé RBM Bijlsma JWJ Burmester GR Dougados M Kerschbaumer A et al. EULAR recommendations for the management of rheumatoid arthritis with synthetic and biological disease-modifying antirheumatic drugs: 2019 update. Ann Rheum Dis. (2020) 79:685–99. 10.1136/annrheumdis-2019-216655 31969328

[16] Gossec L Baraliakos X Kerschbaumer A de Wit M McInnes I Dougados M et al. EULAR recommendations for the management of psoriatic arthritis with pharmacological therapies: 2019 update. Ann Rheum Dis. (2020) 79:700–12. 10.1136/annrheumdis-2020-217159 32434812 PMC7286048

[17] Ward MM Deodhar A Gensler LS Dubreuil M Yu D Khan MA et al. (2019). Update of the American College of Rheumatology/Spondylitis Association of America/Spondyloarthritis Research and treatment network recommendations for the treatment of ankylosing spondylitis and nonradiographic axial spondyloarthritis. Arthritis Care Res (Hoboken). (2019) 71:1285–99. 10.1002/acr.24025 31436026 PMC6764857

[18] Ding Y Huang B Wang Y Hou J Chi Y Zhou Z et al. Janus kinase inhibitor significantly improved rash and muscle strength in juvenile dermatomyositis. Ann Rheum Dis. (2020) 80:543–5. 10.1136/annrheumdis-2020-218582 33115762 PMC7958081

[19] Sanchez GAM Reinhardt A Ramsey S Wittkowski H Hashkes PJ Berkun Y et al. JAK1/2 inhibition with baricitinib in the treatment of autoinflammatory interferonopathies. J Clin Invest. (2018) 128:3041–52. 10.1172/JCI98814 29649002 PMC6026004

[20] Brunner H Synoverska O Ting T Abud Mendoza C Spindler A Vyzhga Y et al. Y, V. Tofacitinib for the Treatment of Polyarticular Course Juvenile Idiopathic Arthritis: Results of a Phase 3 Randomized, Double-blind, Placebo-controlled Withdrawal Study [abstract]. Arthritis Rheumatol. (2019). p. 71. Available online at: https://acrabstracts.org/abstract/tofacitinib-for-the-treatment-of-polyarticular-course-juvenile-idiopathic-arthritis-results-of-a-phase-3-randomized-double-blind-placebo-controlled-withdrawal-study/ (accessed January 5, 2022).

[21] Fraenkel L Bathon JM England BR St Clair EW Arayssi T Carandang K et al. (2021). American College of Rheumatology Guideline for the Treatment of Rheumatoid Arthritis. Arthritis Rheumatol. (2021) 73:1108–23. 10.1016/S0140-6736(21)01255-1 34101376

[22] Ma Y Meng J Jia J Wang M Teng J Zhu D et al. Current and emerging biological therapy in adult-onset Still's disease. Rheumatology (Oxford). (2021) 60:3986–4000. 10.1093/rheumatology/keab485 34117886 PMC8410009

[23] Hu Q Wang M Jia J Teng J Chi H Liu T et al. Tofacitinib in refractory adult-onset Still's disease: 14 cases from a single centre in China. Ann Rheum Dis. (2020) 79:842–4. 10.1136/annrheumdis-2019-216699 32079571 PMC7286046

[24] Huang Z Lee PY Yao X Zheng S Li T. Tofacitinib Treatment of Refractory Systemic Juvenile Idiopathic Arthritis. Pediatrics. (2019) 143:e20182845. 10.1542/peds.2018-2845 30948682

[25] Gillard L Mitrovic S Pouchot J Cohen F Michaud M Reumaux H et al. JAK Inhibitors in Refractory Adult and Childhood-Onset Still's Disease [abstract]. Arthritis Rheumatol. (2021). p. 73. Available onlilne at: https://acrabstracts.org/abstract/jak-inhibitors-in-refractory-adult-and-childhood-onset-stills-disease/. (accessed January 5, 2022).

[26] Tronconi E Miniaci A Pession A. The autoimmune burden in juvenile idiopathic arthritis. Ital J Pediatr. (2017) 43:56. 10.1186/s13052-017-0373-9 28615030 PMC5471888

[27] Conic RR Tamashunas NL Damiani G Fabbrocini G Cantelli M Bergfeld WF. Comorbidities in pediatric alopecia areata. J Eur Acad Dermatol Venereol. (2020). 10.1111/jdv.16727 32531131

[28] Dai YX Chen CC. Tofacitinib therapy for children with severe alopecia areata. J Am Acad Dermatol. (2019) 80:1164–6. 10.1016/j.jaad.2018.12.041 30630026

[29] Jerjen R Meah N Trindade de Carvalho L Wall D Eisman S Sinclair R. Treatment of alopecia areata in pre-adolescent children with oral tofacitinib: A retrospective study. Pediatr Dermatol. (2020) 00:1–6. 10.1111/pde.14422 33099833

[30] Bayart CB DeNiro KL Brichta L Craiglow BG Sidbury R. Topical Janus kinase inhibitors for the treatment of pediatric alopecia areata. J Am Acad Dermatol. (2017) 77:167–70. 10.1016/j.jaad.2017.03.024 28619556

[31] Baechler EC Bilgic H Reed AM. Type I interferon pathway in adult and juvenile dermatomyositis. Arthritis Res Ther. (2011) 13:249. 10.1186/ar3531 22192711 PMC3334651

[32] Paik JJ Casciola-Rosen L Shin JY Albayda J Tiniakou E Leung DG et al. Study of tofacitinib in refractory dermatomyositis: an open-label pilot study of ten patients. Arthritis Rheumatol. (2021) 73:858–65. 10.1002/art.41602 33258553 PMC8084900

[33] Yu ZX Song HM. Toward a better understanding of type I interferonopathies: a brief summary, update and beyond. World J Pediatr. (2020) 16:44–51. 10.1007/s12519-019-00273-z 31377974

[34] Sönmez HE Karaaslan C de Jesus AA Batu ED Anlar B Sözeri B et al. A clinical score to guide in decision making for monogenic type I IFNopathies. Pediatr Res. (2020) 87:745–52. 10.1038/s41390-019-0614-2 31641281 PMC8425764

[35] Boyadzhiev M Marinov L Boyadzhiev V Iotova V Aksentijevich I Hambleton S. Disease course and treatment effects of a JAK inhibitor in a patient with CANDLE syndrome. Pediatr Rheumatol Online J. (2019) 17:19. 10.1186/s12969-019-0322-9 31046790 PMC6498627

[36] Clarke SLN Robertson L Rice GI Seabra L Hilliard TN Crow YJ et al. Type 1 interferonopathy presenting as juvenile idiopathic arthritis with interstitial lung disease: report of a new phenotype. Pediatr Rheumatol Online J. (2020) 18:37. PMID: 32398023; PMCID: PMC7218611. 10.1186/s12969-020-00425-w 32398023 PMC7218611

[37] Balci S Ekinci RMK de Jesus AA Goldbach-Mansky R Yilmaz M. Baricitinib experience on STING-associated vasculopathy with onset in infancy: A representative case from Turkey. Clin Immunol. (2020) 212:108273. 10.1016/j.clim.2019.108273 31626957

[38] Briand C Frémond ML Bessis D Carbasse A Rice GI Bondet V. Efficacy of JAK1/2 inhibition in the treatment of chilblain lupus due to TREX1 deficiency. Ann Rheum Dis. (2019) 78:431–3. 10.1136/annrheumdis-2018-214037 30282666

[39] McLellan KE Martin N Davidson JE Cordeiro N Oates BD Neven B et al. JAK 1/2 Blockade in MDA5 Gain-of-Function. J Clin Immunol. (2018) 38:844–6. 10.1007/s10875-018-0563-2 30443754

[40] Gómez-Arias PJ Gómez-García F Hernández-Parada J Montilla-López AM Ruano J Parra-Peralbo E. Efficacy and safety of janus kinase inhibitors in type I interferon-mediated monogenic autoinflammatory disorders: a scoping review. Dermatol Ther (Heidelb). (2021) 11:733–50. 10.1007/s13555-021-00517-9 33856640 PMC8163936

[41] Berends SE Strik AS Löwenberg M D'Haens GR Mathôt RAA. Clinical Pharmacokinetic and Pharmacodynamic Considerations in the Treatment of Ulcerative Colitis. Clin Pharmacokinet. (2019) 58:15–37. 10.1007/s40262-018-0676-z 29752633 PMC6326086

[42] López-Sanromán A Esplugues JV Domènech E. Pharmacology and safety of tofacitinib in ulcerative colitis. Gastroenterol Hepatol. (2021) 44:39–48. 10.1016/j.gastre.2020.04.007 32829958

